# Total Lesion Glycolysis Improves Tumor Burden Evaluation and Risk Assessment at Diagnosis in Hodgkin Lymphoma

**DOI:** 10.3390/jcm10194396

**Published:** 2021-09-26

**Authors:** Ines Herraez, Leyre Bento, Jaume Daumal, Alessandra Repetto, Raquel Del Campo, Sandra Perez, Rafael Ramos, Javier Ibarra, Francesc Mestre, Joan Bargay, Paloma Lopez, Joan Garcias-Ladaria, Antonia Sampol, Antonio Gutierrez

**Affiliations:** 1 Department of Hematology, Son Llatzer University Hospital, 07198 Palma de Mallorca, Spain; ines.herraez@hsll.es (I.H.); rcampo@hsll.es (R.D.C.); jbargay@hsll.es (J.B.); 2 Institut d’Investigació Sanitària Illes Balears (IdISBa), Son Espases University Hospital, 07120 Palma de Mallorca, Spain; leyre.bento@ssib.es (L.B.); jaume.daumal@gmail.com (J.D.); alessandra.repetto@ssib.es (A.R.); sandra.perez@ssib.es (S.P.); rafaelf.ramos@ssib.es (R.R.); jibarra@hsll.es (J.I.); franciscoj.mestre@ssib.es (F.M.); paloma.lopez@ssib.es (P.L.); jgarcias@ssib.es (J.G.-L.); antonia.sampolm@ssib.es (A.S.); 3 Department of Hematology, Son Espases University Hospital, 07120 Palma de Mallorca, Spain; 4 Department of Nuclear Medicine, Son Espases University Hospital, 07120 Palma de Mallorca, Spain; 5 Department of Pathology, Son Espases University Hospital, 07120 Palma de Mallorca, Spain; 6 Department of Pathology, Son Llatzer University Hospital, 07198 Palma de Mallorca, Spain; 7 Department of Radiotherapy, Son Espases University Hospital, 07120 Palma de Mallorca, Spain; 8 Department of Radiology, Son Espases University Hospital, 07120 Palma de Mallorca, Spain; 9 Department of Dermatology, Son Espases University Hospital, 07120 Palma de Mallorca, Spain

**Keywords:** Hodgkin lymphoma, Ann Arbor stage, metabolic tumor volume, total lesion glycolysis, risk assessment, survival

## Abstract

Hodgkin lymphoma (HL) is a hematological malignancy with an excellent prognosis. However, we still need to identify those patients that could experience failed standard frontline chemotherapy. Tumor burden evaluation and standard decisions are based on Ann Arbor (AA) staging, but this approach may be insufficient in predicting outcomes. We aim to study new ways to assess tumor burden through volume-based PET parameters to improve the risk assessment of HL patients. We retrospectively analyzed 101 patients with HL from two hospitals in the Balearic Islands between 2011 and 2018. Higher metabolic tumor volume (MTV) and total lesion glycolysis (TLG) were significantly associated with a higher incidence of III-IV AA stages, B-symptoms, hypoalbuminemia, lymphopenia, and higher IPS. Standardized uptake value (SUVmax) was significantly related to AA stage and hypoalbuminemia. We found that TLG or the combination of SUVmax, TLG, and MTV significantly improved the risk assessment when compared to AA staging. We conclude that TLG is the best single PET/CT-related tumor-load parameter that significantly improves HL risk assessment when compared to AA staging. If confirmed in a larger and validated sample, this information could be used to modify standard frontline therapy and justifies the inclusion of TLG inside an HL prognostic score.

## 1. Introduction

Hodgkin lymphoma (HL) is a hematological malignancy formed by malignant cells, so-called Reed–Sternberg cells (RSC), surrounded by an inflammatory microenvironment of reactive cells. A high percentage of patients are cured with conventional strategies, but approximately 15–30% relapse or progress. The standard tool to assess disease burden is the Ann Arbor (AA) staging that classically categorizes HL in I to IV stages, considering the number of affected lymph nodes and/or extranodal sites and their location related to diaphragm as well as the presence or absence of B symptoms [1]. However, AA staging lacks accuracy in predicting outcome [2,3].

New ways to assess tumor burden, such as baseline fluorodeoxyglucose positron emission tomography/computed tomography (FDG PET/CT), detect active disease with higher sensitivity in comparison with computed tomography (CT) [2,4]. Standardized uptake value (SUV) is the most frequent semiquantitative PET metric used for measuring tumor glucose metabolism. It is defined as the ratio of the decay-corrected FDG concentration in a volume of interest (VOI) to the injected dose normalized to the patient’s body weight. SUVmax is defined as the maximum value of SUV in a VOI representing the highest metabolism in the tumor, and it is commonly used in response criteria in PET scans after treatment in oncology [5].

Metabolic tumor volume (MTV) and total lesion glycolysis (TLG) are volume-based PET parameters, and they reflect tumor biology. MTV represents the volume (mL or cm^3^) resulting from the sum of the metabolic volume of each tumor tissue with increased threshold FDG uptake. TLG is defined as the product of the average SUV (SUVmean) of the total tumor multiplied by the corresponding MTV; it represents both the tumor size and the extent of FDG uptake and is representative of the metabolic activity throughout the entire tumor (including both RSC and its inflammatory microenvironment). 

Until now, many studies have described a prognostic role of these parameters in non-Hodgkin lymphoma (NHL) [3,6,7,8,9,10,11] and HL [12,13,14,15,16,17,18], some of them comparing PET-based assessment with standard AA staging and the specific prognostic indexes [19]. However, current standard tools to assess HL tumor burden and prognosis at diagnosis are still based only on AA staging [2,20]. We aim to explore ways to improve disease burden testing using FDG PET/CT-related parameters to better stratify HL patients at the time of diagnosis.

## 2. Materials and Methods

### 2.1. Sample Selection

We retrospectively included patients with HL who were homogeneously treated with adriamycin, bleomycin, vinblastine, and dacarbazine (ABVD) +/− radiotherapy (RT) at Son Espases and Son Llatzer University Hospitals in Palma de Mallorca from the databases of Pharmacy, Pathology, and Nuclear Medicine Departments to avoid selection bias. Those patients treated with different schemes were excluded. In our centers, in general, radiotherapy is administered to the following cases: patients with I-II AA stages without risk factors ((European Organization for Research and Treatment of Cancer (EORTC) or German Hodgkin Score Group (GHSG) < 1)) with low risk of toxicity in the involved area might choose between 2 cycles of ABVD plus radiotherapy or 4 cycles of ABVD; patients with I–II AA stages with risk factors of EORTC or GHSG might choose between 4 cycles of ABVD plus radiotherapy or 6 cycles of ABVD; patients with III–IV stages received radiotherapy only if localized partial response (PR) in the interim or final PET/CT. No patient received escalated or de-escalated chemotherapy according to the interim PET result.

Standard clinical prognostic variables were obtained from medical records (age, gender, stage, bulky, and Eastern Cooperative Oncology Group Performance Status (ECOG PS), and main prognostic scores, International Prognostic Score (IPS), EORTC, and GHSG, were calculated [20,21]. This study was approved by the Ethics Committee of Balearic Islands with the number IB4071/19.

### 2.2. Assessment of PET-Related Parameters

FDG PET/CT was done at baseline, interim (24 h before or the same day of the second or third cycle), and 21–28 days after the end of the treatment. Response assessment was performed following Deauville’s criteria [22]. All patients were instructed to fast for at least 4 to 6 h before injection of FDG. Blood glucose level was measured before the scan to ensure it was less than 180 mg/dL before FDG administration. Whole-body PET was acquired using a dedicated PET/CT system (General Electric Discovery ST 16). PET scans were performed from the proximal femur to the base of the skull (to ensure the bladder was at its most empty). They were acquired in 3D mode (2 min/bed) with reconstruction with iterative method 55 to 65 min after intravenous administration of 3.7 MBq/Kg of 18F-FDG. Low-dose non-contrast-enhanced CT scans were used for anatomic registration and attenuation correction. 

MTV and TLG were measured using the semiautomatic software plugin Beth Israel Fiji [23] (shareware from the Beth Israel Deaconess Medical Center, Division of Nuclear Medicine and Molecular Imaging available at http://sourceforge.net/projects/bifijiplugins/, accessed on 18 August 2021). A region of interest (ROI) was automatically drawn around each pathological focus of FDG uptake. In each ROI, voxels presenting a 41% maximum standardized uptake value (SUVmax) threshold were incorporated to define the MTV, as recommended by the European Association of Nuclear Medicine [24]. Then we reviewed automatic ROIs and deleted false-positive delineations (for example, delineations that include brain, kidney, or bladder physiological uptakes), and moreover, it was possible to add additional manual ROIs for missing uptake area (for example, small or low uptake can be missed by the automatic segmentation). All PET/CT in this work were centrally reviewed and performed in the same Nuclear Medicine Department using the same software and hardware. Additionally, all evaluations were examined by two experts.

Extra-nodal involvement was considered in the volume calculation according to the following rules: the liver, lung, and bone marrow were considered involved only in cases of focal uptake; homogeneous bone marrow uptake was not included in the tumor volume; and spleen involvement was considered in cases of focal uptake or diffuse uptake higher than 150% of the liver background [25,26].

### 2.3. Statistical Methods

We used receiver operating curves (ROC) analysis to obtain the optimal cutoff for progression or death of all experimental FDG PET/CT-related variables. Variables following binomial distributions (i.e., response rate) were expressed as frequencies and percentages. Comparisons between qualitative variables were done using the Fisher Exact Test or chi-square. Comparisons between quantitative and qualitative variables were performed through non-parametric tests (Mann–Whitney U-test or Kruskal–Wallis). 

Time to event variables, overall survival (OS), and progression-free survival (PFS) were measured from the date of therapy onset and were estimated according to the Kaplan–Meier method. Comparisons between the variables of interest were performed by the log-rank test. PFS was considered the time from diagnosis to disease progression or death of any cause. All *p*-values reported were 2-sided, and statistical significance was defined at *p* < 0.05. To analyze and compare the risk assessment ability of the biomarkers, we used concordance probability estimates (CPE) and c-index. 

## 3. Results

### 3.1. Characteristics of the Patients

A total number of 101 patients with classic HL homogeneously treated with ABVD +/− RT were retrospectively analyzed at the time of diagnosis at Son Espases (n = 61) and Son Llatzer (n = 40) University Hospitals in Palma de Mallorca between August 2011 to November 2018. Presenting features of the patients are shown in Table 1. 

Briefly, the median age was 37 years (14–83 years), 53% of patients had an III–IV AA stages, and 10% had bulky disease. Treatment was as follows: 83% of I–II AA stages without risk factors (EORTC or GHSG < 1) were treated with two to four cycles of ABVD with RT in 48%. A total of 71% of I–II AA stages with risk factors (EORTC or GHSG > 1) and 94% of III–IV AA stages were treated with six cycles of ABVD. All I–II AA stages with risk factors that received two to four cycles of ABVD were consolidated with RT as well as 7% of III–IV AA stages. 

### 3.2. Response to Therapy and Survival

Most patients obtained a complete response (CR) to ABVD induction therapy (n = 85; 84%) and three PR (3%) for an overall response rate of 87%. Only 10 patients (10%) failed frontline therapy, and three patients (3%) died from toxicity before the first response assessment. With a median follow-up of 45 months (11–90), four-year PFS and OS were 78% (CI95%: 69–87) and 92% (CI95%: 86–98), respectively. Table 2 shows the univariate analysis of the influence of main standard prognostic factors in survival. 

### 3.3. Analysis of the Baseline FDG PET/CT Parameters

The optimal cutoffs obtained for MTV, TLG, and SUVmax were 32.5 (mL), 167.8, and 10.4, respectively. We studied the relationship between main prognostic factors in HL and metrics parameters of FDG PET/CT. Higher MTV and TLG were significantly associated with a higher incidence of III–IV AA stages but also with the presence of B-symptoms, hypoalbuminemia, lymphopenia, and higher IPS scores. SUVmax was significantly related to AA stage and hypoalbuminemia (Table 3). 

**Figure 1:**
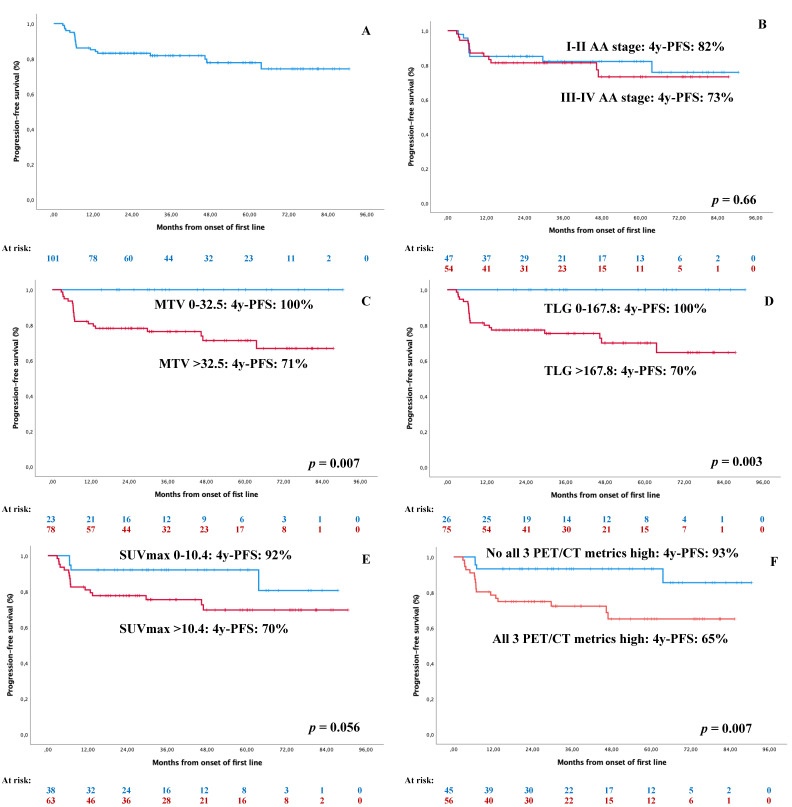
Impact on PFS of standard and PET/CT-related tumor-load assessments. (**A**) PFS of the whole series; (**B**) PFS according to AA staging; (**C**) PFS according to MTV; (**D**) PFS according to TLG; (**E**) PFS according to SUVmax; (**F**) PFS according to all three PET/CT metrics.

**Figure 2:**
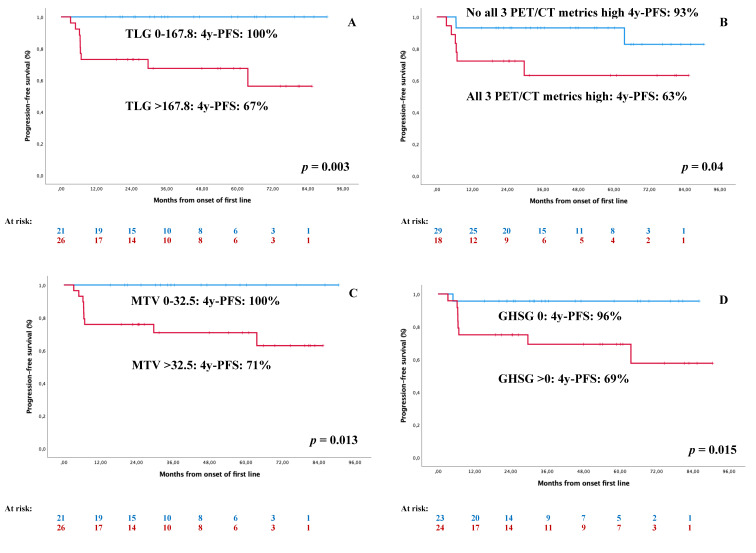
Impact on PFS of GHSG and PET/CT-related tumor-load assessments in I–II AA stages. (**A**) PFS according to TLG; (**B**) PFS according to all three PET/CT metrics; (**C**) PFS according to MTV; (**D**) PFS according to GHSG at diagnosis.

Considering tumoral load assessment, Table 4 shows the impact on survival of standard AA staging and new FDG PET/CT variables. In the univariate survival analysis, PFS was significantly influenced by MTV (*p* = 0.007) and TLG (*p* = 0.003) but not the AA stage (Table 4; Figure 1). Figure 2 shows the impact of the standard GHSG prognostic score, as well as the PET/CT metrics in stage I–II HL.

As shown in Table 4, these three FDG PET/CT parameters had a better risk assessment than standard AA staging, being able to differentiate two risk groups with 93% and 65% 4y-PFS (Figure 1F) together with 96% and 88% 4y-OS, respectively. Using CPE, we analyzed the risk assessment provided by any of the measures of tumor load: the standard AA stage (0.56), MTV (0.68), TLG (0.69), SUVmax (0.61), and the combination of all three PET/CT parameters (0.72). There was a significant improvement in the risk assessment provided by TLG (*p* = 0.032) and all three PET/CT parameters (*p* = 0.035) when compared to the standard AA stage using c-index. 

We explored the sensitivity and specificity to predict frontline treatment failure (progression or death of any cause). Sensitivity was 100%, 100%, 81%, 57%, and 26% for MTV, TLG, SUVmax, AA staging, and interim PET/CT, respectively. Specificity was 29%, 32%, 42%, 47%, and 93%, respectively. Positive predictive value was 27%, 28%, 27%, 22%, and 50%. Negative predictive value was 100%, 100%, 89%, 81%, and 83%. In other words, FDG PET/CT parameters were able to identify at diagnosis most patients failing or not to standard ABVD frontline therapy (Figure 1) and did so much better than AA staging or interim PET/CT. However, interim PET/CT showed a superior specificity. 

## 4. Discussion

HL has an excellent outcome with standard chemotherapy, but a small subset of patients will eventually relapse or progress. Detecting these refractory patients at the time of diagnosis is very important to modify the initial treatment approach. The first task after the diagnosis of malignancy is precisely quantifying the tumor load, which normally correlates with the prognosis of the disease. Until now, treatment decisions in HL regarding tumor load are mainly based on the AA stage [27]. The presence of bulky disease and the number of regions involved in I–II AA stages is considered an adverse prognostic factor in the two staging systems used for localized HL: EORTC and GHSG [28,29,30,31]. Initially, these indirect surrogates of tumor burden were measured by CT; however, in the last years, FDG PET/CT replaced CT, as it better reflects active disease or extranodal sites involvement [2,4].

Furthermore, quantitative parameters, such as MTV, TLG, and SUVmax, were obtained from FDG PET/CT. These PET/CT-related parameters have been studied both at baseline or as part of interim or final disease assessments after therapy. It has been described that MTV could have a prognostic role in HL [14,15,32] and non-Hodgkin lymphoma [6,7]. However, data on TLG in HL is scarcer.

In NHL, some studies concluded that high levels of MTV and TLG at diagnosis significantly predicted a poor OS and shorter PFS also in combination with other scores, such as the Prognostic Index for T-cell lymphoma (PIT), International Prognostic Index (IPI) in extranodal NK/T-cell lymphoma, or Follicular Lymphoma International Prognostic Index (FLIPI) [6,7,33]. In diffuse, large B-cell lymphoma (DLBCL), a study showed that MTV was a better parameter than the AA staging system for predicting outcome in patients treated with R-CHOP [3].

Three studies in HL concluded that MTV and TLG were related to PFS and OS in early stages, improving risk stratification and identifying those patients with a higher risk of progression [13,14,15]. In a cohort of 59 HL patients, Kanoun et al. showed that patients with a low MTV at baseline had a significantly better four-year PFS than those with a high MTV (85 % vs. 42 %, *p* = 0.001). In a multivariate analysis, only the variation of SUVmax in PET2 and baseline MTV remained independent predictors of PFS (*p* = 0.0005 with HR 6.4, and *p* < 0.007, HR 4.2, respectively), and tumor bulk did not reach statistical significance [15]. In advanced HL treated with eBEACOPP, interim MTV showed to be a prognostic factor [17]. Additionally, volumetric parameters could predict outcomes in relapsed/refractory disease, improving the predictive power of the pretransplant FDG PET/CT [12,34]. In pediatric HL patients, MTV at diagnosis was shown to predict inadequate response to induction therapy better than other FDG-PET parameters [32].

We found that TLG or the combination of the three PET/CT metrics are the only PET/CT-related parameters that significantly improved the risk assessment when compared to AA staging. The combination of the three PET/CT metrics only slightly improved the CPEs obtained by TLG (0.72 vs. 0.69, respectively), showing AA staging yields a much lower risk assessment (CPE: 0.56). Our results show that patients with higher MTV and TLG have a bigger tumor burden and a more aggressive malignancy that may increase the risk of failing frontline therapy with ABVD, as observed with a worse PFS. Obviously, this may be eventually translated to OS, although fortunately, many HL patients respond to second or further lines of therapy.

To our knowledge, our manuscript is the first one reporting that TLG is the best single PET/CT-related tumor-load parameter that significantly improves HL risk assessment when compared to AA staging. However, several works identified some of these metrics as significantly related to response or survival and important in providing risk assessment in HL. Rogasch et al. concluded in a pediatric HL retrospective analysis that high total MTV best predicted inadequate response to standard therapy [32]. In elderly HL patients, Albano et al. concluded that SUVmax is an independent prognostic factor for OS and PFS, while MTV and TLG were only for PFS [18]. Another study in early-stage HL patients by Akhtari et al. concluded that MTV and TLG could reclassify early unfavorable HL patients, predicting those that will have worse outcomes [14]. Finally, Pike et al., in an oral presentation, concluded that in advanced HL patients, TLG could be a strong independent risk factor for prognosis, predicting those patients that will need intensive therapy [35].

It is important to mention that PET/CT-related metrics need optimal standardization. Adams, MC et al. reviewed all the factors that can potentially affect the reproducibility of SUV measurements. They provided recommendations on ways to minimize them. They distinguished between biological and technological factors. Following these recommendations, we minimized biological factors, measuring blood glucose level before each scan and avoiding scanning if it was more than 180 mg/dL; using lean body mass to minimize any weight dependence of SUV; and acquiring it at the same post-injection time. Considering technological factors, we reduced quantitation variability, as we used the same PET scan with the same acquisition and reconstruction parameters; we did not use contrast material for PET/CT, avoiding incorrect attenuation correction; quality control and calibration are usually performed before each scan; dose calibrator and PET scanner clocks are constantly synchronized; and finally, we measured radioactivity in the syringe before and after injection of the radiotracer. Finally, the semi-automatic process of ROIs delineation was systematically and centrally reviewed by two experts [36].

TLG includes the information provided by MTV but multiplied by the average SUV of the total tumor, representing both the tumor size and the extend of FDG uptake, which could improve the risk assessment [5]. The outstanding sensitivity and negative predictive value of the TLG, specially in early HL cases, provide a more precise staging and improve risk assessment. Another interesting point is the way of calculation the cutoff for PET/CT metrics in HL. Most of them used the median or specific percentiles (such as 80th) [14,17,26]. However, this approach may be biased by the specific characteristics of any particular sample. For this reason, it should be preferred to use ROC curves, which allow linking the cutoff to the particular event that we may wish to predict [14,16]. Again, our work is the first one presenting a ROC cutoff for TLG predicting PFS in HL. For this reason, our TLG cutoff is much lower than the one used in other works based on the median or percentiles (167.8 vs. 1703) [14]. A ROC-based cutoff is more prone to be useful in clinical practice, although it should also be obtained using a big multicentric consensus sample or standardized for any particular center or hardware. At the same time, this ROC-based cutoff should be tested in new or even old clinical trials in HL in order to confirm its value as well as be validated in independent cohorts. Future clinical trials could use this information to select those patient candidates to receive new drugs, different approaches, or even de-escalation of therapy.

The main limitations of our study include a short sample, the retrospective nature of the work, and the absence of discovery and validation samples. For these reasons, our results should be confirmed in a larger and validated sample. Currently, we are planning to perform a validation of these results in a large, multicentric cohort.

## 5. Conclusions

We conclude that TLG and the combination of the three PET/CT-related quantitative metrics of tumor burden (MTV, TLG, and SUVmax) obtained at diagnosis, in HL, could be valuable tools to better stratify the risk when compared with standard AA staging. Furthermore, TLG combines information on the amount of tumor burden as well as its metabolic activity. In our series, TLG, MTV, and the combination of all three PET/CT-related metrics were able to identify which patients were going to fail or not regarding the standard frontline ABVD regimen. This information provides a more precise staging and improves risk assessment, so it could be used to modify the standard approach at the time of frontline therapy as well be included inside prognostic scores for HL. Again, clinical trials will be needed to confirm and prove its value.

## Figures and Tables

**Table 1:** Characteristics of patients.

Median Age (Range)	37 (14–83)
Age> 45:	32 (31%)
Age > 60:	17 (17%)
Gender: M/F	61 (60%)/40 (40%)
Ann Arbor stage:	
I–II	47 (46%)
III–IV	54 (53%)
B symptoms:	42 (42%)
Bulky:	10 (10%)
ECOG PS:	
0–1	63 (62%)
>1	38 (38%)
Albumin < 4 g/dL	49 (50%)
Hb < 10.5 g/dL	49 (50%)
Leucocytes ≥ 15,000/microL	12 (12%)
Lymphopenia < 600/microL or <8%	16 (16%)
Elevated ESR (I–II AA stages)	20 (43%)
More than 3 nodal sites (I–II AA stages)	12 (39%)
More than 4 nodal sites (I–II AA stages)	3 (10%)
GHSG > 0:	78 (77%)
EORTC > 0:	72 (71%)
IPS:	
0–3	82 (81%)
>3	19 (19%)
Radiotherapy:	22 (22%)
Median baseline MTV (range)	95.4 (3.1–912.7)
Median baseline TLG (range)	528.5 (7.4–5167.6)
Median baseline SUVmax (range)	11.9 (1.7–23.5)

M, male; F, female; ECOG PS, Eastern Cooperative Oncology Group Performance Status; Hb, hemoglobin; ESR, erythrocyte sedimentation rate; GSHG, German Hodgkin Study Group; EORTC, European Organization for Research and Treatment of Cancer; IPS, International prognostic score; MTV, metabolic tumor volume; TLG, total lesion glycolysis; SUVmax, maximum standardized uptake value.

**Table 2:** Survival analysis according to main standard prognostic variables in HL.

	4y-PFS (95%CI)	*p*	4y-OS	*p*
Age:		0.063		<0.001
0–44 years	82% (77–87)		100% (NA)	
>44 year	67% (58–79)		74% (65–83)	
Age:		0.06		<0.001
0–60 years	82% (77–86)		97% (94–99)	
>60 years	47% (26–68)		67% (54–80)	
Sex:		0.98		0.52
Male	79% (73–84)		91% (87–95)	
Female	76% (68–84)		94% (90–98)	
AA stage:		0.66		0.39
I–II	82% (76–88)		96% (93–99)	
III–IV	73% (66–80)		88% (83–93)	
B-symptoms:		0.27		0.81
Yes	69% (60–79)		93% (87–98)	
No	82% (77–87)		91% (87–95)	
Bulky:		0.45		0.91
Yes	70% (55–84)		86% (72–99)	
No	78% (73–83)		93% (90–96)	
ECOG PS:		0.54		0.76
0–1	79% (73–85)		91% (88–95)	
>1	79% (72–85)		95% (91–98)	
Albumin:		0.18		0.019
<4	73% (66–80)		83% (77–89)	
≥4	80% (74–87)		100% (NA)	
Hb:		0.79		0.2
<10.5 g/dL	71% (54–88)		100% (NA)	
≥10.5 g/dL	78% (73–83)		90% (87–94)	
Leucocytes:		0.32		0.46
≥15,000/microL	75% (62–87)		91% (82–100)	
<15,000/microL	78% (73–83)		92% (89–95)	
Lymphocytes:		0.2		0.43
<600/microL and <8%	69% (57–80)		93% (86–100)	
>600/microL or <8%	80% (75–85)		92% (88–95)	
IPS (whole series):		0.18		0.6
0–3	80% (75–85)		91% (88–95)	
>3	66% (51–80)		94% (88–100)	
IPS (III–IV AA stages):		0.81		0.61
0–3	75% (67–83)		86% (79–93)	
>3	66% (49–82)		93% (87–100)	
ESR (I-II AA stages):		0.015		0.57
Normal	91% (85–97)		100% (NA)	
Elevated	70% (60–80)		90% (83–97)	
Number of nodal sites (I–II AA stages):		0.018		0.49
0–2	95% (90–100)		95% (87–100)	
3 or more	67% (53–80)		92% (88–100)	
Number of nodal sites (I–II AA stages):		0.51		0.41
0–3	86% (79–92)		96% (93–100)	
4 or more	67% (39–94)		67% (39–94)	

AA stage, Ann Arbor stage; ECOG PS, Eastern Cooperative Oncology Group Performance Status; Hb, hemoglobin; IPS, International prognostic score; ESR, erythrocyte sedimentation rate; PFS, progression-free survival; 95%CI, 95% confidence interval; OS, overall survival; NA, not available.

**Table 3:** Relationship between FDG PET/CT variables and main prognostic factors in HL.

	All Patients	MTV			TLG			SUVmax		
	N = 101	Low	High	*p*	Low	High	*p*	Low	High	*p*
Median age (range)	37 (14–83)	33 (15–83)	37 (14–82)	0.66	30 (14–83)	39 (15–82)	0.31	36 (14–83)	37 (15–82)	0.96
Age > 45:	32 (31%)	8 (35%)	24 (31%)	0.8	7 (27%)	25 (33%)	0.63	12 (32%)	20 (32%)	1
Age > 60:	17 (17%)	3 (13%)	14 (18%)	0.81	3 (11%)	14 (19%)	0.59	6 (16%)	11 (17%)	1
Gender: M/F	61 (60%)/40 (40%)	11 (48%)/12 (52%)	50 (64%)/28 (36%)	0.22	14 (54%)/12 (46%)	47 (63%)/28 (37%)	0.49	21 (55%)/17 (45%)	40 (63%)/23 (56%)	0.53
AA stage:				0.002			<0.001			0.044
I	1 (1%)	1 (4%)	0 (0%)		1 (4%)	0 (0%)		1 (3%)	0 (0%)	
II	46 (45%)	17 (74%)	29 (37%)		20 (77%)	26 (35%)		23 (60%)	23 (36%)	
III	27 (27%)	4 (17%)	23 (29%)		5 (19%)	22 (29%)		8 (21%)	19 (30%)	
IV	27 (27%)	1 (4%)	26 (33%)		0 (%)	27 (36%)		6 (16%)	21 (33%)	
AA stage:				0.001			<0.001			0.013
I–II	47 (46%)	18(78%)	29 (37%)		21 (81%)	26 (35%)		24 (63%)	23 (36%)	
III–IV	54 (53%)	5 (22%)	49 (63%)		5 (19%)	49 (65%)		14 (37%)	40 (63%)	
B-symptoms:	42 (42%)	3 (13%)	39 (50%)	0.002	4 (15%)	38 (51%)	0.002	11 (29%)	31 (49%)	0.061
Bulky:	10 (10%)	1 (4%)	9 (11%)	0.54	0 (0%)	10 (13%)	0.11	3 (8%)	7 (11%)	0.86
ECOG PS > 1	38 (38%)	6 (26%)	32 (41%)	0.23	6 (23%)	32 (43%)	0.1	12 (32%)	26 (41%)	0.4
Albumin < 4 g/dL	49 (50%)	3 (14%)	46 (61%)	<0.001	3 (13%)	46 (62%)	<0.001	10 (29%)	39 (62%)	0.003
Hb < 10.5 g/dL	49 (50%)	1 (4%)	17 (22%)	0.11	1 (4%)	17 (23%)	0.062	5 (13%)	13 (21%)	0.43
Leucocytes ≥ 15,000/microL	12 (12%)	0 (0%)	12 (15%)	0.1	0 (0%)	12 (16%)	0.069	1 (3%)	11 (17%)	0.06
Lymphopenia < 600/microL or <8%	16 (16%)	0 (0%)	16 (20%)	0.041	0 (0%)	16 (21%)	0.024	3 (8%)	13 (21%)	0.1
IPS:				0.02			0.011	5 (13%)	14 (22%)	0.3
0–3	82 (81%)	23 (100%)	59 (76%)		26 (100%)	56 (75%)				
>3	19 (19%)	0 (0%)	19 (24%)		0 (0%)	19 (25%)				

MTV, metabolic tumor volume; TLG, total lesion glycolysis; SUVmax, maximum standardized uptake value; M, male; F, female; AA stage, Ann Arbor stage; ECOG PS, Eastern Cooperative Oncology Group Performance Status; Hb, hemoglobin; IPS, International prognostic score.

**Table 4:** PFS and OS according to tumoral load variables.

	N	4y-PFS	*p*	4y-OS	*p*
AA stage:			0.55		0.77
I	1	100% (NA)		100% (NA)	
II	46	82% (76–88)		96% (92–99)	
III	27	85% (78–92)		88% (82–95)	
IV	27	58% (45–72)		88% (79–96)	
AA stage:			0.18		0.97
I–III	74	83% (79–88)		93% (90–96)	
IV	27	58% (45–72)		88% (79–96)	
MTV:			0.007		0.11
0–32.5	23	100% (NA)		100% (NA)	
>32.5	78	71% (65–77)		89% (86–93)	
TLG:			0.003		0.07
0–167.8	26	100% (NA)		100% (NA)	
>167.8	75	70% (64–76)		89% (85–93)	
SUVmax:			0.056		0.13
0–10.4	38	92% (88–96)		94% (89–100)	
>10.4	63	70% (63–76)		90% (86–94)	
PET/CT parameters:			0.008		0.090
0–1	26	100% (NA)		100% (NA)	
2	19	84% (76–93)		90% (80–99)	
3	56	65% (58–72)		88% (84–93)	
PET/CT parameters:			0.007		0.035
0–2	45	93% (90–97)		96% (92–100)	
3	56	65% (58–72)		88% (84–93)	

PFS, progression-free survival; OS, overall survival; AA stage, Ann Arbor stage; MTV, metabolic tumor volume; TLG, total lesion glycolysis; SUVmax, maximum standardized uptake value; PET/CT, positron emission tomography/computed tomography; NA, not available.

## Data Availability

Data are available upon direct request from the corresponding author.
